# Diet in Disease

**Published:** 1893-06-10

**Authors:** Ernest Hart

**Affiliations:** Bachelier-ès-Sciences-es-Lettres (rèstreint), formerly Student of the Faculty of Medicine of Paris, and of the London School of Medicine for Women


### Diet in Disease.

By Mrs. Ernest Hart, Bachelier-ès-Sciences-es-Lettres (rèstreint), formerly Student of the Faculty of Medicine of Paris, and of the London School of Medicine for Women.

### XXII.—UNDER-FEEDING AND OVER-EATING (*Continued*).

**In Advanced Life.**—It may seem hard for the man, who in youth has known the pinch of poverty who remembers how the cut of mutton, with a supply of potatoes and greens, scarcely sufficed for a vigorous appetite, should find that in the prosperity of later life an eight-course dinner of delicacies fails to tempt him, and that nevertheless, his physician warns him that the attack of gout from which he is suffering means that he is eating too much, and that his diet must be lowered. Is life, then, never to give satifactions? Must youth know hunger and old age satiety? Must the poor muscle-worker never have enough food to give energy to his frame, and must the rich idler have so much to eat that disease is the consequence? To find the happy mean, to live according to sweet reasonableness and knowledge, is the aim of the teachings of science, and if to these are added the principles of Christian communism, the wealth of later life will not lead to self-indulgence, but to the mitigation of the sufferings of those who want the means of life. Of this result, one knows many splendid examples. I recall one of a gentleman, now in possession of a very large income, who told me that in his youth he lived on a salary of 10s. a week. He early made up his mind that to eat little and drink less would be his rule in life. To this resolution he has adhered, though fortune has come to him. Nearly an octogenarian, he is still a man of untiring vigour of body and mind. Simple in life, he dispenses his great fortune as a custodian for his Master, while living amid the refinement and cultured surroundings of an English gentleman.

In advanced life tissue change is slow, digestion is less active, and the ability to assimilate food is greatly diminished. As middle-age is passed and old age approaches, the intake of food, particularly of nitrogenous and fatty foods, must be steadily diminished. Sir Henry Thompson, who has written forcibly on this subject, says: "As we increase in age, less energy and activity remain, and less expenditure can be made, less power to eliminate at fifty than at thirty, still less at sixty and upwards. Less nutriment must, therefore, be taken in proportion as age advances, or rather as activity diminishes, or the individual will suffer. If he continues to consume the same abundant breakfasts, substantial lunches, and heavy dinners, which at the summit of his power he could dispose of almost with impunity, he will in time either accumulate fat, or become acquainted with gout and rheumatism, or show signs of unhealthy deposit of some kind in some part of the body, processes which must inevitably empoison, undermine, or shorten his remaining term of life. He must reduce his intake because a smaller expenditure is an enforced condition of existence. At seventy the man's power is still further diminished, and the nutriment must correspond thereto if he desires still another term of comfortable life. And why should he not? Then at eighty, with less activity, there must be still less 'support.' And on this principle he may yet long continue to live."* The kind of food which the elderly should particularly diminish are the nitrogenous and fatty varieties. Growth has ended, tissue change is slow, the energy which induced activity is gone, and nitrogen is no longer required to build up, after the ceaseless wear and destruction of the body. To persist in taking nitrogenous or meaty foods after middle-age is passed, is to throw a burden on the kidneys which they are not able of bearing, and the diseases of gout, rheumatism, renal cirrhosis, and apoplexy are the result. In old age the power of fasting is not so great as in earlier life, and the meals while being smaller, should therefore be more frequent, the intervals between them being short. A small amount of alcohol with food is also often beneficial with the aged. The long fast of the night, during which sleep is not sound, is ill borne and a glass of milk or a cup of beef tea may often be taken in the night with advantage. It is difficult to lay down any fixed rules for the dieting of the old, for age is not according to length of years, but to the number of infirmities. We all know men of seventy-five who are as active, physically and mentally, as others of sixty. Sir Henry Thompson's rule is the best, namely, to diminish the intake of food as activity diminishes. As age increases, let the quantity taken be less, and let fish and poultry take the place of butcher's meat, and farinaceous foods of highly-flavoured dishes. Saccharin will be found a useful substitute for sugar, and cream for oily fatty foods. A nourishing stimulant to be highly recommended, and which may be taken between meals, is an ounce of dry cherry brandy mixed with a wineglassful of cream.

Sir George Humphry has investigated the life-histories of centenarians in England with the view of ascertaining the causes and circumstances of longevity. The report was published by the Collective Investigation Committee of the British Medical Association in 1887. As one reads of the habits and life of these men and women who attained to the age of one hundred years and more, one is struck by the fact that they were almost invariably lean people, of spare habit, and of great moderation in eating and drinking. Of thirty-seven, three took no animal food, four took very little, twenty a little, ten a moderate amount, and only one acknowledged taking much meat. With regard to alcohol, the returns are much the same, and abstemiousness is found to be the rule of life of these centenarians. Fifteen had been total abstainers, either during the whole or part of their lives; two took very little alcohol, twenty-two a little, and ten a moderate amount. Sir George Humphry's interesting and valuable collection of facts regarding centenarians confirms opinions which have been held from time to time by various persons, in opposition to the generally accepted view that as age increases and strength diminishes food should be more stimulating and strengthening. The most remarkable of these persons was Cornaro, an Italian nobleman, who lived in the fifteenth and sixteenth centuries, and who attained the

*"Diet in Relation to Age and Activity." By Sir Henry Thompson.

age of upwards of 100 years. He seems in middle life to have suffered from dyspepsia, brought on by over-indulgence, for he says that he had "fallen into different kinds of disorders, such as pains in my stomach, and often stitches, and spices of the gout, attended by what was almost still worse, an almost continual slow fever, a stomach generally out of order, and a perpetual thirst." At the age of forty he decided that abstemiousness and regularity should be the order of his life, instead of the previous course of indulgence in eating and drinking, which was surely driving him to his grave. He kept his resolution for a year, at the end of which time he declared himself free of all his complaints. He states that his rule was to take as much food and wine as would check appetite without completely satisfying it. "I accustomed myself," he says, "to contrive matters so as never to cloy my stomach with eating and drinking; but constantly to rise from the table with a disposition to eat and drink still more. . . What with bread, meat, the yelk of an egg, and soup, I ate as much as weighed in all 12 oz., neither more nor less. I drank in all 14 oz of wine." Cornaro lived on this meagre diet to a vigorous old age. He wrote several treatises on the subject of diet, urging others to follow his example; one of these was written when he had attained the age of ninety-five, and shows that he was in full possession of his faculties.

Though the too liberal use of meat by those who live sedentary lives and who are past middle age is strongly deprecated, and though it is a fact that beans and grains can furnish a large supply of albuminous food, yet there is an abundance of evidence in support of the opinion that no diet is so favourable to the production of that condition of the muscles which enables a man to undergo prolonged and excessive muscular exertion as lean meat, particularly beef. Under this diet the muscles seem to attain a firmness and contractile power not otherwise produced. During training the diet consists of underdone meat, a small amount of bread and vegetables; fluids are restricted, and only a small quantity of tea and beer are allowed; all sweets, pastry, puddings, entrées, sauces, pickles, and condiments are strictly forbidden. This diet, accompanied with exercise, will in about the space of six weeks reduce all superfluous fat, and give the muscles firmness, bulk, and great contractile power. In countries where continuous physical exertion is the necessity of life, the race has generally discovered for itself, without the teaching of science, the great value of a meat diet. Thus, in the limitless plains of the Pampas, which can only be traversed on horseback, the Indians have learnt by experience that meat alone will give them the muscular force to gallop all day long on horseback. Sir Francis Head, in his account of his "Journeys Across the Pampas," tells how he could not stand the fatigue of constant galloping, but was obliged, after five or six hours' riding, to rest in a carriage till he had adopted the diet of the Indians, and lived on beef and water. "But after," he says, "I had been riding for three or four months, and had lived on beef and water, I found myself in a condition which I can only describe by saying that I felt no exertion could kill me." Vegetable feeders may be and are capable of great feats of strength; but the capacity to endure prolonged physical exertion belongs to the meat-eater. The gentleman may dispense with butcher's meat without harm; the navvy and miner require beef and mutton. In fact, in this topsy-turvy world the underfed are the poor working men, who need food whereby to work; and the overfed are the well-to-do middle-aged, who should be abstemious in order to enjoy the good things with which their lives abound. A community of goods might be to the benefit of both.

